# Great Plasticity in a Great Pathogen: Capsular Types, Virulence Factors and Biofilm Formation in ESBL-Producing *Klebsiella pneumoniae* from Pediatric Infections in Uruguay

**DOI:** 10.3390/antibiotics13020170

**Published:** 2024-02-09

**Authors:** Lucía Araújo, Romina Papa-Ezdra, Pablo Ávila, Victoria Iribarnegaray, Inés Bado, Hector Telechea, Virginia Garcia-Fulgueiras, Rafael Vignoli

**Affiliations:** 1 Departamento de Bacteriología y Virología, Instituto de Higiene, Facultad de Medicina, Universidad de la República, Montevideo 11600, Uruguay; lu.araujo.87@gmail.com (L.A.); rpapa@higiene.edu.uy (R.P.-E.); pablo1915@gmail.com (P.Á.); ibado@higiene.edu.uy (I.B.); 2 Departamento de Microbiología, Instituto de Investigaciones Biológicas Clemente Estable, Montevideo 11600, Uruguay; victoria.iribarnegaray@gmail.com; 3 Departamento de Patobiología, Facultad de Veterinaria, Universidad de la República, Montevideo 12100, Uruguay; 4 Unidad Cuidados Intensivos Pediátricos, Facultad de Medicina, Universidad de la República, Montevideo 11600, Uruguay; hmteleo@gmail.com

**Keywords:** *Klebsiella pneumoniae*, ESBL, K-types, *fyuA*, *kfuBC*, biofilm, ST, MDR, pediatric-infections

## Abstract

*Klebsiella pneumoniae* is widely recognized as an opportunistic hospital and community pathogen. It is one of the priority microorganisms included in the ESKAPE group, and its antibiotic resistance related to extended-spectrum β-lactamases (ESBL) is a global public health concern. The multi-drug resistance (MDR) phenotype, in combination with pathogenicity factors, could enhance the ability of this pathogen to cause clinical infections. The aim of this study was to characterize pathogenicity factors and biofilm formation in ESBL-producing *K. pneumoniae* from pediatric clinical infections. Capsular types, virulence factors, and sequence types were characterized by PCR. Biofilm formation was determined by a semiquantitative microtiter technique. MDR phenotype and statistical analysis were performed. The K24 capsular type (27%), virulence factors related to iron uptake *fyuA* (35%) and *kfuBC* (27%), and sequence types ST14 (18%) and ST45 (18%) were the most frequently detected. Most of the strains were biofilm producers: weak (22%), moderate (22%), or strong (12%). In 62% of the strains, an MDR phenotype was detected. Strains with K24 capsular type showed an association with ST45 and the presence of *fyuA*; strains with *kfuBC* showed an association with moderate or strong biofilm production and belonging to ST14. Weak or no biofilm producers were associated with the absence of *kfuBC*. The MDR phenotype was associated with the main ESBL gene, *bla*_CTX-M-15_. The high plasticity of *K. pneumoniae* to acquire an MDR phenotype, in combination with the factors exposed in this report, could make it even more difficult to achieve a good clinical outcome with the available therapeutics.

## 1. Introduction

*Klebsiella pneumoniae* is one of the priority pathogens included in the ESKAPE group [1], among the most challenging microorganisms to treat. It belongs to the critical group on the WHO priority pathogens list for research and development of new antibiotics [2]. Recent studies have positioned this microorganism among the top five agents most associated with mortality worldwide, regardless of its susceptibility to antibiotics [3]. Additionally, data from the Antimicrobial Resistance Collaborators group underscore the significance of pathogens responsible for more than 250,000 deaths associated with antimicrobial resistance (AMR) in 2019, where *K. pneumoniae* ranked third [4].

*K. pneumoniae* is the causative agent of a wide range of infections, including pneumonia, bacteremia and sepsis, meningitis, pyogenic liver abscesses, and urinary tract infections, among others [5]. This microorganism is well-known as an opportunistic hospital and community pathogen, and the multi-drug resistance (MDR) phenotype it exhibits is a major global public health concern. The evolution of MDR in *K. pneumoniae* is largely driven by the acquisition of AMR genes, which are particularly prevalent among globally spread clones [6,7,8] and are often responsible for hospital outbreaks. Currently, AMR in this pathogen is mostly related to carbapenem and third-generation cephalosporin resistance [4]. Different extended-spectrum β-lactamases (ESBL) conferring resistance to third-generation cephalosporins have been reported in our country, including those from the main CTX-M groups (CTX-M-15, CTX-M-2, CTX-M-9 and CTX-M-14, and CTX-M-8 [9,10,11]), SHV-derived enzymes (SHV-2 and SHV-5 [9,10,11]), and carbapenemases conferring resistance to carbapenems (KPC-2 and NDM-1 [12]). Additionally, resistance to fluoroquinolones due to plasmid-mediated quinolone resistance (PMQR) mechanisms QnrA and QnrB, principally, and aminoglycosides Aac(6′)Ib due to modifying enzymes have also been detected [9,10,11,12]. Due to the high plasticity of this pathogen, in some cases, ESBL, carbapenemases, PMQR, and aminoglycosides modifying enzymes may coexist in the same clinical strain, leading to limited treatment options for patients involved [12]. 

In addition to AMR, the ability to cause serious infections in *K. pneumoniae* isolates is associated with other relevant factors, including capsular types (K-types), virulence factors, biofilm formation, sequence types, and clonal distribution, among the most significant [5,6,13,14,15]. 

In relation to K-types, there are more than 130 predicted types, such as K2 (https://bigsdb.pasteur.fr/klebsiella/, accessed on 13 December 2023), which are associated with strains causing invasive infections; K2 is particularly considered antiphagocytic and serum-resistant [6]. The genes responsible for capsule production in *K. pneumoniae* are located in a chromosomal operon containing several genes involved in capsule production. Within this operon, the *wzi* gene encodes a surface protein involved in capsule attachment to the outer membrane. K-antigen typing is a combination of serological methods and sequencing of the *wzi* gene, where different *wzi* locus sequences are associated with specific K-antigens [5]. 

Other virulence factors related to the pathogenicity of this microorganism include fimbrial and non-fimbrial adhesins, iron-scavenging systems, and surface polysaccharides (capsule, as we mentioned). In relation to fimbrial adhesins, type 1 (*fimH* gene) and 3 fimbriae (*mrkD* gene) are the major adhesive structures characterized as pathogenicity factors [5]. In terms of iron-scavenging systems, several siderophores are expressed in *K. pneumoniae*, including enterobactin, yersiniabactin, salmochelin, and aerobactin [5]. 

The capability of biofilm formation is another relevant pathogenic trait that some isolates may possess. In the report conducted by Zheng J.X. and colleagues, the association between type 1 and 3 fimbriae with the ability to enhance biofilm formation on urinary catheters is analyzed [14]. 

Among the different isolates, the identification of sequence types and clonal distribution is important. There are well-recognized successful clones harboring an MDR phenotype, such as ST11 and ST258, or ST14, ST15, ST17, and ST37, which have been associated with worldwide outbreaks in humans in recent years [15]. 

The aim of this exploratory study was to investigate pathogenicity factors and biofilm formation in ESBL-producing *K. pneumoniae* from pediatric clinical infections.

## 2. Results 

### 2.1. Isolates

A total of 40 ESBL-producing *K. pneumoniae* isolates were characterized in two study periods [9,10]. The most frequently detected associations of resistance mechanisms were *bla*_CTX-M-15_/*aac(6′)Ib-cr*/*qnrB* (n = 14), *bla*_CTX-M-2_/*aac(6′)Ib* (n = 7), and *bla*_SHV-5_/*aac(6′)Ib* (n = 5) (Table 1).

### 2.2. K-Types, Virulence Factors, and Hypermucoviscosity

We characterized 17 different K-types in the studied collection. The most frequent K-type was K24 in 11/40 strains. K2 was found in only 4/40 isolates (Table 1).

The virulence factors *mrkD*, *wabG,* and *ureA* were identified in all strains. Only one strain was negative for *fimH* or *uge*, strain 26 was *fimH* negative, and strain 30 was *uge* negative (data not shown). The *fyuA* gene was present in 14/40 strains, and the *kfuBC* gene was present in 11/40 strains (Table 1). The genes *magA*, *allS*, *rmpA*, *cf29a*, *iutA,* and *chuA* were not identified in the collection. 

The string test was negative, and none of the isolates was considered to be a hypermucoviscous isolate. 

### 2.3. Biofilm

**Figure 1:**
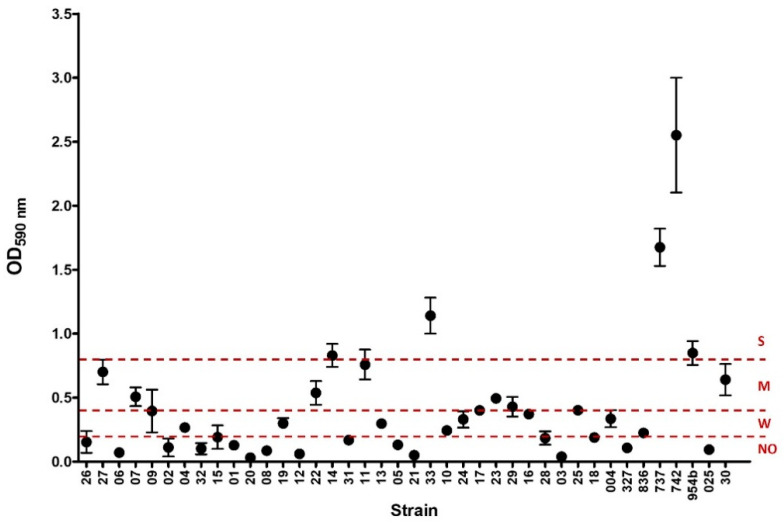
Biofilm formation of the 40 *K. pneumoniae* strains studied. The graphic shows the values (mean and standard deviation) of optical density at 590 nm (OD590 nm) of crystal violet obtained for each strain. Dashed lines at 0.2, 0.4, and 0.8 express the threshold value for each biofilm capability formation category: no biofilm producer (NO), weak (W), and moderate (M) biofilm, respectively. Values above 0.8 were considered as strong (S) biofilm formation.

Biofilm formation was detected in 23/40 of the strains, which were categorized as strong (n = 5), moderate (n = 9), or weak (n = 9) biofilm producers (Table 1, Figure 1). A wide variety of K-types were detected among biofilm producers, and none of them prevailed. Most of the strong biofilm producers were positive for the ABC iron transport system *kfuBC*, and only one was also positive for the yersiniabactin receptor *fyuA*. Three of the involved isolates came from invasive samples (blood). In moderate biofilm producers, only three isolates were positive for *kfuBC*, and one of them was also positive for *fyuA*; most of the strains in this group were obtained from urine samples. In relation to isolates defined as weak biofilm producers, *fyuA* was more prevalent than in the other groups. The strains involved here were obtained from either invasive or non-invasive samples. One strain was positive for *fyuA*/*kfuBC* and was recovered from a cerebrospinal fluid sample. No biofilm producers accumulated most of the *fyuA*-positive strains. In terms of ESBL production, we identified *bla*_CTX-M-15_ among the isolates that were not biofilm producers in almost half of the cases. However, in the other groups, none ESBL prevailed (Table 1). 

### 2.4. Sequence Types and Pulse Types

**Figure 2:**
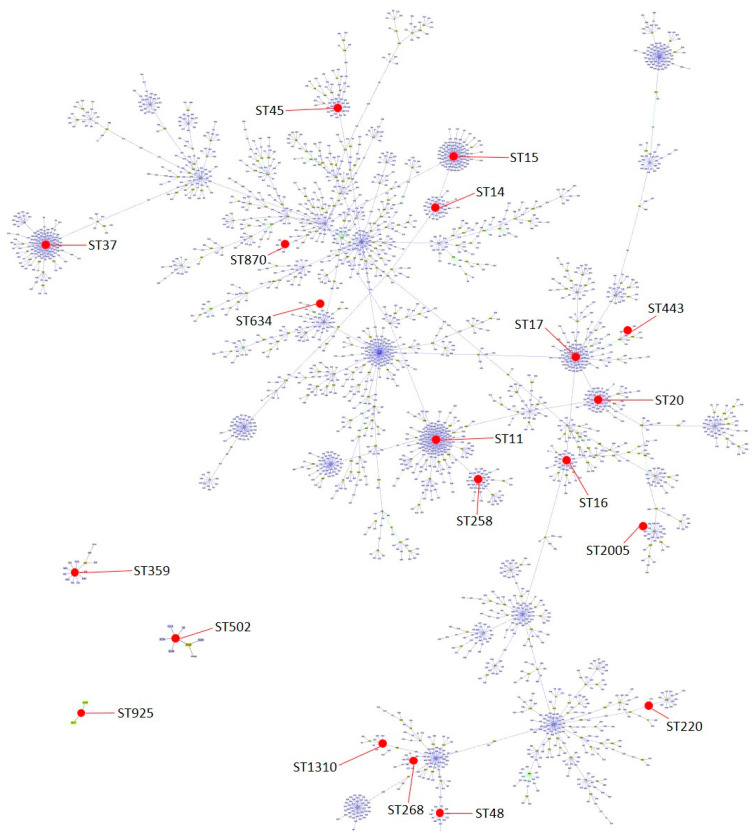
Population snapshot of *K. pneumoniae* with groups defined at single locus variant (SLV) level and indicating the distribution of the 20 sequence types found in this work (represented in red dots). Snapshot generated by goeBURST v.1.2.1 software using a dataset downloaded from https://bigsdb.pasteur.fr/klebsiella/ (accessed on 13 December 2023).

A total of 20 different STs were detected, with the most frequently being ST14 (n = 7) and ST45 (n = 7); 13/20 of the identified STs were represented by only one isolate (Table 1). Global optimal eBURST analysis revealed a great phylogenetic diversity and a scattered distribution among the characterized STs in this report. However, some of them are related to each other. For example, ST258 is a single locus variant (SLV) of ST11, ST14 and ST15 are SLVs of each other and triple locus variants of ST11, ST16 and ST20 are SLVs of ST17, and ST443 is a double locus variant of ST17 (Figure 2).

Six out of seven of the ST14 isolates were categorized as moderate or strong biofilm producers, whereas the same proportion, but in the absence of biofilm production, was detected in ST45 (n = 6). In ST14, K2 isolates were the most frequent, and the presence of the ABC iron transport system *kfuBC* was detected in three cases related to invasive infections (blood or cerebrospinal fluid). All ST45 strains belonged to K24 K-type, with *fyuA* being positive in many of these strains, and urine was the main source (Table 1). 

The UPGMA analysis of PFGE resulted in 28 different pulse types (PT), data not shown. Most PTs (n = 21) were represented by a single isolate, and no dominant PT was detected (Table 1).

### 2.5. Antibiotic Susceptibility

The MDR phenotype was found in 25/40 strains when data were analyzed using either EUCAST-2012 or EUCAST-2023 guidelines. The MIC_50_, MIC_90,_ and susceptibility values were interpreted for each antibiotic (Table 2). Amikacin resistance increased from 10% (n = 4) to 52% (n = 21) when the data were analyzed using the latter guidelines. (Table 1). 

### 2.6. Statistical Results

We statistically investigated significant associations (*p* < 0.05) for the main variables in each group (Table 3).

Isolates from the K24 capsular type were associated with the presence of the yersiniabactin receptor *fyuA* and were related to ST45. The presence of the virulence factor *fyuA* was associated with isolates from ST45 and with isolates harboring *bla*_CTX-M-15_. Strains positive for the ABC iron transport system *kfuBC* were more likely to be strong or moderate biofilm producers and being from ST14; weak or no biofilm producers were associated with the absence of *kfuBC*. Being a strong or moderate biofilm producer was associated with ST14. Finally, an MDR phenotype was associated with the main ESBL gene *bla*_CTX-M-15_, while *bla*_CTX-M-2_ was associated with strains not exhibiting an MDR phenotype (Table 3). The statistical results remained consistent when data were interpreted according to EUCAST-2023.

When we analyzed associations between K24, virulence factors (*kfuBC*/*fyuA*), biofilm production (strong/moderate), sequence types (ST14/ST45), ESBL type (*bla*_CTX-M-15_/*bla*_CTX-M-2_/*bla*_SHV-5_) or MDR phenotype and invasive samples (blood/cerebrospinal fluid/synovial fluid), we found none (Table 3).

## 3. Discussion

In both study periods, ESBL-producing *K. pneumoniae* was the main EPE detected: 40% [9] and 43% [10], respectively. 

The wide variety of capsular types characterized is in accordance with the polyclonal distribution of the studied isolates, not being able to identify any predominant pulse type. K24 was the most frequent K-type. Some epidemiological surveys have suggested that K24 is commonly associated with ST15 [16] and a carbapenem-resistant phenotype in *K. pneumoniae* strains [17]. Nevertheless, we found only one ST15 among K24 producers, while the remaining K24 strains belonged to ST14 or ST45. In this sense, K24 isolates showed a statistical association with ST45, a sequence type considered an established global AMR high-risk clone among ESBL-producing *K. pneumoniae* isolates [18,19]. 

In relation to the characterization of iron-scavenging systems, *fyuA* was associated with ST45 isolates and with the ESBL gene *bla*_CTX-M-15_ in the involved strains. Additionally, more than half of *fyuA*-positive cases were of K24 type. This virulence factor was related to ST25/K2 or ST45/K62 in *K. pneumoniae* harboring *bla*_CTX-M-15_ in Mexico [20] and to carbapenem-resistant strains in Italy [21]. The *kfuBC* gene was found in 27% of the studied isolates; in 55% of the cases, the isolates were recovered from invasive samples. According to the literature, this virulence factor is more frequently found in invasive clinical strains (from liver abscess, meningitis, or endophthalmitis) than in non-invasive strains [5,22,23]. The presence of *kfuBC* was associated with the capability of being a strong or moderate biofilm producer and being from ST14. K-types in *kfuBC* positive strains were K2 (ST14), K17 (ST870), K24 (ST14, ST15 and ST45) and *wzi231* (ST925). In these cases, ESBL involved were in order of frequency: *bla*_CTX-M-15_, *bla*_CTX-M-8_, *bla*_CTX-M-9_, *bla*_SHV-5,_ and *bla*_SHV-2_. Public databases exhibit some different results; Lev A.I. and colleagues detected *kfuBC* in K2 isolates from ST65 or ST2174 with *bla*_SHV-11_ [24], while other reports, including Mukherjee S. and colleagues, found *kfuBC* in K2 isolates from ST14 with *bla*_CTX-M-15_ [25]. 

Regarding K2-type isolates, all of them were from ST14 and identified as strong or moderate biofilm producers. In 3/4 cases, the isolates were recovered from invasive samples (blood and cerebrospinal fluid), and all of them harbored *kfuBC*. Our results are consistent with other works that report ESBL-producing *K. pneumoniae* K2 isolates from ST14 in different countries, sometimes with *kfuBC*, as mentioned previously [25,26,27]. It is important to emphasize that invasive K2 isolates exhibiting strong/moderate biofilm formation could acquire additional antibiotic resistance mechanisms, such as carbapenemases, making the achievement of successful outcomes even more challenging.

Regarding sequence types from the first study period, it is noteworthy that we identified important globally distributed clones such as ST15 and other important recognized international outbreak clones like ST20 [15]. However, only one isolate was detected in each case and was not present in the next study period.

In reports from our country, the globally distributed clone ST258 is more frequently associated with ESBL-harboring isolates rather than carbapenemase producers [10,11]. In this work, the strains ST258 harboring *bla*_CTX-M-15_ or *bla*_SHV-5_ were characterized as single clones, in weak/no biofilm producers and without *kfuBC* or *fyuA* virulence factors. 

According to antibiotype, MDR phenotype was found in 62% of the isolates studied regardless of the guideline used. This percentage aligns with the range reported in other studies involving clinical isolates of Enterobacterales [28,29]. The presence of the MDR phenotype was associated with the main ESBL gene characterized, *bla*_CTX-M-15_. CTX-M-15 is an enzyme frequently reported in *K. pneumoniae* worldwide [30] and in our region in particular [11,12,31,32]. MIC_50_, MIC_90,_ and resistance values were high for all antibiotics (except for amikacin) when EUCAST-2012 breakpoints were used. It is interesting that amikacin resistance increased from 10% to 52% when data were analyzed with the latest breakpoints due to the lower values for interpreting the resistance profile in EUCAST-2023 guidelines; this could result in fewer treatment options when ESBL-producing *K. pneumoniae* is isolated or suspected. The high resistance values for the analyzed antibiotics are related to the co-existence of different antibiotic resistance mechanisms within the same *K. pneumoniae* isolate. We characterized different combinations of genes arrays in the same isolate: *bla*_CTX-M-15_; *aac(6′)Ib*, *aadA,* and *B* alleles; *aac(6′)Ib-cr*, *qnrA,* and *B* variants; and *dfr* types and class 1 integrons, conferring resistance to third-generation cephalosporins, aminoglycosides, fluoroquinolones, and trimethoprim-sulfamethoxazole, respectively [9,12]. It is important to remark that trimethoprim-sulfamethoxazole could be an appropriate treatment option in the cases of urine isolates with MIC values of 40 mg/L. This antibiotic is recommended for a wide range of infections, including urinary tract infections, respiratory infections, and enteric infections (https://www.eucast.org/ accessed on 13 January 2024).

Although we were expecting to find some particular K-type, virulence factor, biofilm production (strong or moderate), sequence type, or ESBL type when the sample came from an invasive infection, we did not find any relation in this sense. This could be related to the small number of samples and also to some clinical reasons like comorbidities (for example, immunosuppression) or the nature of some infections (for example, nosocomial ones).

## 4. Materials and Methods

### 4.1. Sample Collection

ESBL-producing *K. pneumoniae* were collected and processed in the “Hospital Pediátrico Centro Hospitalario Pereira Rossell” (HP-CHPR) microbiology laboratory from two study periods: 7/20 ESBL-producing Enterobacterales (EPE) from 2009 [9] and 33/77 EPE from 2010 to 2012 [10].

### 4.2. K-Types, Virulence Factors, and Hypermucoviscosity Characterization

K-types were defined according to the *wzi* alleles by comparing the partial 447 bp sequences of this gene with the *K. pneumoniae wzi* database (https://bigsdb.pasteur.fr/klebsiella/ accessed on 13 December 2023). If the designated *wzi* allelic type correlated with a single capsular serotype in the database, the serotype was used for the description of the capsular types (e.g., K2, K17). If it correlated with multiple or non-capsular serotypes, the *wzi* allelic type was used (e.g., *wzi50*, *wzi86*) [33].

Virulence genes for mucoviscosity (*magA*, chromosomal mucoviscosity associated gene A, and *rmpA*, regulator of mucoid phenotype A), biosynthesis of LPS (*uge* and *wabG*), adhesin (non-fimbrial, *cf29a*; fimbrial, *fimH* and *mrkD*), allantoin metabolism (*allS*), ABC iron transport system (*kfuBC*), siderophore (*iutA*, aerobactin receptor; *fyuA*, yersiniabactin receptor; *chuA*, iron-carrying molecules receptor) and urease (*ureA*) were assessed for all isolates by PCR according to Brisse et al. [13].

The hypermucoviscous feature of strains was examined by the string test, as previously described [34].

### 4.3. Biofilm Quantification

Bacterial ability to produce biofilm was assessed using a semiquantitative microtiter technique. Briefly, the strains were grown overnight in Luria Bertani (LB) broth at 37 °C under static conditions. Twenty microliters from this culture were inoculated into 180 μL of LB in 96-well flat-bottomed polystyrene microtiter plates and then incubated for 48 h at 37 °C without shaking. Planktonic bacteria were removed, and the attached bacteria were washed three times with phosphate-buffered saline (PBS). The plates were stained with 1% crystal violet (CV) for 15 min at room temperature. Then, the excess dye was removed with three subsequent PBS washes, and CV was solubilized with 200 μL of 95% ethanol. The biofilm biomass was measured by optical density at 590 nm (OD590) using a Microplate Reader (Varioskan, Thermo Scientific). Strains were classified as follows: OD ≤ ODc = no biofilm producer; ODc < OD ≤ (2 × ODc) = weak biofilm producer; (2 × ODc) < OD ≤ (4 × ODc) = moderate biofilm producer; and (4 × ODc) < OD = strong biofilm producer; where ODc were control wells with media without bacteria [35].

### 4.4. Pulsed-Field Gel Electrophoresis (PFGE) and Multilocus Sequence Typing (MLST) Characterization

For strains collected in the first period, *XbaI*-PFGE to detect pulse types (PT) and MLST to detect sequence types (ST) were performed [10]. Sequence typing was conducted using the guidelines described in the *K. pneumoniae* MLST database (https://bigsdb.pasteur.fr/klebsiella/ accessed on 13 December 2023). Both PFGE and MLST analyses for the strains collected in the second period were previously performed and published [10]. PFGE results from both periods (n = 40) were interpreted using the unweighted pair-group method with an arithmetic mean (UPGMA), and the ST distribution for all strains was visualized with the global optimal eBURST [36] using the dataset available at the MLST database (https://bigsdb.pasteur.fr/klebsiella/ accessed on 13 December 2023).

### 4.5. Antibiotic Susceptibility Testing

Antibiotic susceptibility testing was performed using a VITEK^®^2 Compact system (bioMérieux) [9,10]. Data related to AMR were interpreted using EUCAST guidelines (2012 vs 2023, https://www.eucast.org/ accessed on 13 December 2023). MDR phenotype was defined as isolates exhibiting resistance to at least one agent in three or more of the following antibiotic families: third-generation cephalosporins, aminoglycosides (amikacin or gentamicin), fluoroquinolones (ciprofloxacin), and folate pathway antagonists (trimethoprim-sulfamethoxazole) [37]. MIC_50_, MIC_90,_ and the percentage of susceptibility were determined for each antibiotic. 

### 4.6. Statistical Analysis

We analyzed associations between the different variables studied: capsular types, virulence factors, biofilm production, sequence types, ESBL, MDR phenotype, and type of infection. Nominal variables were compared using the Chi-square test for variables divided into categories [38]. A two-tailed *p*-value less than 0.05 was considered statistically significant. Statistical analyses were performed using SPSS 23.0 software (IBM SPSS Inc., Chicago, IL, USA). The associations were investigated with the aim of generating future hypotheses for studies with a more clinical approach. 

## 5. Conclusions

Among the great diversity we were able to detect between the factors analyzed in ESBL-producing *K. pneumoniae* in this report, we were able to highlight interesting findings. We characterized the most frequent K-type, K24, in association with isolates belonging to ST45 and the presence of the *fyuA* yersiniabactin receptor. Isolates with the virulence factor *kfuBC* (ABC iron transport system) showed an association with the capability of being a strong or moderate biofilm producer and belonging to ST14. Remarkably, strains that were weak or had no biofilm producers were associated with the absence of *kfuBC*. It might be possible that *kfuBC* plays a role in moderate or strong biofilm formation. The MDR phenotype was high, and it was associated with the main ESBL gene, *bla*_CTX-M-15_. The high prevalence of the MDR phenotype could be partially explained by the additional resistance mechanisms [*aac(6′)Ib-cr*/*qnrB*] in isolates harboring *bla*_CTX-M-15_.

We believe that future clinical research is important to evaluate the association between the different clinical presentations and factors studied in this report. 

Despite our collection consisting of only 40 isolates from many years ago, it is worth highlighting that the results presented here represent the first findings on this topic in our country. These findings could contribute to our understanding of the characteristics of ESBL-producing *K. pneumoniae* clinical infections in the pediatric population.

The high plasticity of *K. pneumoniae* emphasizes the importance of continuous surveillance, which could result not only in better use of available therapeutic resources but also in the control of this microorganism in hospital settings.

## Figures and Tables

**Table 1:** Main features of *K. pneumoniae* isolates. Data are sorted in increasing order by K-type/*wzi*.

									MIC (mg/L)						
Number	Sample	Resistance Mechanisms	K/*wzi*	*kfuBC*	*fyuA*	BF	ST	PT	CTX	CAZ	AK	GN	CIP	SXT	Antibiotype
11	csf	*bla*_CTX-M-15_/*aac(6′)Ib-cr*/*qnrB*	K2	+	−	M	14	XVII	≥64	16	8	≥16	2	≥320	3GC/GN/CIP/SXT
14	blood	*bla*_CTX-M-15_/*aac(6′)Ib-cr*/*qnrB*	K2	+	−	S	14	XII	≥64	16	4	≥16	2	≥320	3GC/GN/CIP/SXT
29	urine	*bla*_CTX-M-8_/*aac(6′)Ib*	K2	−	−	M	14	XVII	32	≤1	16	8	1	≤20	3GC/GN/CIP */AK *
737	blood	*bla*_CTX-M-8_/*aac(6′)Ib*/*qnrB*	K2	+	−	S	14	XXV	16	≤1	16	8	1.5	≤20	3GC/GN/CIP/AK *
16	sw	*bla*_SHV-5_	K17	+	−	W	870	VIII	4	≥64	≤2	≤1	≤0.25	≤20	3GC
17	urine	*bla*_SHV-5_	K17	+	−	M	870	VIII	32	≥64	≤2	≤1	≤0.25	≤20	3GC
18	urine	*bla*_SHV-5_	K17	+	−	NO	870	VIII	4	≥64	≤2	≤1	≤0.25	≤20	3GC
04	blood	*bla*_CTX-M-15_/*aac(6′)Ib-cr*/*qnrB*	K20	−	−	W	268	XVIII	≥64	≥64	8	≥16	2	≤20	3GC/GN/CIP
025	urine	*bla*_CTX-M-2_/*aac(6′)Ib*	K24	−	−	NO	45	XXIII	≥256	4	16	≥16	0.032	≤20	3GC/GN/AK *
05	urine	*bla*_CTX-M-15_/*aac(6′)Ib-cr*/*qnrB*	K24	−	+	NO	45	XV	≥64	16	16	≥16	2	≥320	3GC/GN/CIP/SXT/AK *
06	blood	*bla*_CTX-M-15_/*aac(6′)Ib-cr*/*qnrB*	K24	−	+	NO	45	XIII	≥64	16	16	≥16	2	≥320	3GC/GN/CIP/SXT/AK *
07	blood	*bla*_CTX-M-15_/*aac(6′)Ib-cr*/*qnrB*	K24	+	+	M	14	XI	≥64	16	16	≥16	2	≥320	3GC/GN/CIP/SXT/AK *
08	urine	*bla*_CTX-M-15_/*aac(6′)Ib-cr*/*qnrB*	K24	−	+	NO	45	XV	≥64	8	4	≥16	2	≥320	3GC/GN/CIP/SXT
09	ct	*bla*_CTX-M-15_/*aac(6′)Ib-cr*/*qnrB*	K24	−	+	W	14	XII	≥64	16	4	≥16	2	≥320	3GC/GN/CIP/SXT
12	urine	*bla*_CTX-M-15_/*aac(6′)Ib-cr*/*qnrB*	K24	−	+	NO	45	XV	≥64	8	4	≥16	2	≥320	3GC/GN/CIP/SXT
13	csf	*bla*_CTX-M-15_/*aac(6′)Ib-cr*/*qnrB*	K24	+	+	W	45	X	≥64	16	16	≥16	2	≥320	3GC/GN/CIP/SXT/AK *
327	urine	*bla*_SHV-5_/*aac(6′)Ib*	K24	−	−	NO	45	XXIV	≥256	≥64	12	≤1	0.023	≥320	3GC/SXT/AK *
33	sw	*bla*_SHV-2_	K24	+	+	S	14	XVII	≤1	16	≤2	≤1	≤0.25	≤20	3GC
954b	urine	*bla*_CTX-M-8_	K24	+	−	S	15	XXVIII	64	≤1	1.5	≤1	≥32	≥320	3GC/CIP/SXT
23	urine	*bla*_CTX-M-2_/*aac(6′)Ib*	K27	−	−	M	11	XIX	≥64	16	16	≥16	≥4	80	3GC/GN/CIP/SXT/AK *
836	urine	bla_SHV-2_/*aac(6′)Ib*	K28	−	+	W	20	XXVII	32	≤1	24	8	0.032	≤20	3GC/AK/GN
742	blood	*bla*_SHV-2_	K62	−	−	S	17	XXVI	32	4	16	≤1	0.023	≤20	3GC/AK *
30	urine	*bla*_CTX-M-9_/*aac(6′)Ib*/*qnrA*	*wzi050*	−	−	M	502	I	≥64	≤1	16	8	0.25	≥320	3GC/GN/SXT/AK *
31	urine	*bla*_CTX-M-15_/*aac(6′)Ib-cr*	*wzi050*	−	−	NO	16	II	≥64	≥64	≤2	≤1	≥4	≥320	3GC/CIP/SXT
15	blood	*bla*_SHV-5_	*wzi086*	−	−	NO	1310	VI	≥256	≥64	≤2	≤1	≤0.25	≥320	3GC/SXT
10	blood	*bla*_CTX-M-15_/*aac(6′)Ib-cr*/*qnrB*	*wzi118*	−	+	W	443	XIV	≥64	≥64	16	≥16	2	≥320	3GC/GN/CIP/SXT/AK *
19	urine	*bla*_SHV-5_/*aac(6′)Ib*	*wzi154*	−	−	W	258	IX	8	≥64	≥64	≥16	≥4	40	3GC/AK/GN/CIP
20	blood	*bla*_SHV-5_/*aac(6′)Ib*	*wzi154*	−	−	NO	258	IX	≥256	≥64	≥64	≥16	≥4	40	3GC/AK/GN/CIP
28	blood	*bla*_CTX-M-2_/*aac(6′)Ib*	*wzi154*	−	−	NO	37	VII	≥64	≤1	16	≥16	≤0.25	≤20	3GC/GN/AK *
32	urine	*bla*_CTX-M-15_/*aac(6′)Ib-cr*	*wzi154*	−	−	NO	258	IX	≥64	≥64	≥64	4	≥4	≥320	3GC/AK/CIP/SXT/GN *
01	blood	*bla*_CTX-M-15_/*aac(6′)Ib-cr*/*qnrB*	*wzi167*	−	+	NO	48	XVI	≥64	8	≤2	≥16	2	≥320	3GC/GN/CIP/SXT
02	urine	*bla*_CTX-M-15_/*aac(6′)Ib-cr*/*qnrB*	*wzi167*	−	+	NO	48	XVI	≥64	8	≤2	≥16	2	≥320	3GC/GN/CIP/SXT
03	sw	*bla*_CTX-M-15_/*aac(6′)Ib-cr*/*qnrB*	*wzi167*	−	+	NO	48	XVI	≥64	8	≤2	≥16	2	≥320	3GC/GN/CIP/SXT
22	urine	*bla*_SHV-5_/*aac(6′)Ib*	*wzi177*	−	−	M	220	V	16	≥64	≤2	≥16	0.5	≥320	3GC/GN/SXT
21	urine	*bla*_SHV-5_/*aac(6′)Ib*	*wzi209*	−	+	NO	2005	IV	32	≥64	8	8	0.5	≤20	3GC/GN
004	sf	*bla*_CTX-M-9_/*aac(6′)Ib*	*wzi231*	+	−	W	925	XXII	8	≥64	8	≥16	0.023	≥320	3GC/GN/SXT
24	urine	*bla*_CTX-M-2_/*aac(6′)Ib*	*wzi236*	−	−	W	359	XX	≥64	4	16	≥16	≤0.25	≤20	3GC/GN/AK *
26	urine	*bla*_CTX-M-2_/*aac(6′)Ib*	*wzi236*	−	−	NO	359	XXI	≥64	16	16	≥16	≤0.25	≤20	3GC/GN/AK *
25	urine	*bla*_CTX-M-2_/*aac(6′)Ib*	*wzi281*	−	−	M	634	III	≥64	16	16	≥16	≤0.25	40	3GC/GN/AK *
27	urine	*bla*_CTX-M-2_/*aac(6′)Ib*	*wzi281*	−	−	M	634	III	≥64	4	16	≥16	≤0.25	40	3GC/GN/AK *

Isolates numbers are displayed according to previous reports [9,10]. Abbreviations: csf, cerebrospinal fluid; sw, surgical wounds; ct, catheter tip; sf, synovial fluid; K, K-type; +, presence; −, absence; BF, biofilm; NO, no producer; W, weak producer; M, moderate producer; S, strong producer; ST, sequence type; PT, pulse type; MIC, minimum inhibitory concentration; CTX, cefotaxime; CAZ, ceftazidime; AK, amikacin; GN, gentamicin; CIP, ciprofloxacin; SXT, trimethoprim-sulfamethoxazole; 3GC, third-generation cephalosporins; *, strains resistant to CIP, AK or GN, respectively, according to EUCAST 2023.

**Table 2:** Minimum inhibitory concentration data and antibiotic susceptibility percentages for *K. pneumoniae* isolates.

	Breakpoint ^a^ S (mg/L)	Breakpoint I ^b^ (mg/L)	Breakpoint R (mg/L)	MIC Range (mg/L)	MIC_50_ (mg/L)	MIC_90_ (mg/L)	% S	% I	% R
CTX	≤1	2	≥4	≤1–≥256	≥64	≥64	2	0	98
CAZ	≤1	2–4	≥8	≤1–≥64	16	≥64	15	10	75
AK	≤8	16	≥32	≤2–≥64	16	16	47	43	10
GN	≤2	4	≥8	≤1–≥16	≥16	≥16	23	2	75
CIP	≤0.5	1	≥2	≤0.25–≥32	2	≥4	45	2	53
SXT	≤20	40	≥80	≤20–≥320	≥320	≥320	35	10	55

^a^ according to EUCAST 2012 guidelines; ^b^ was specially considered for this analysis. Abbreviations: MIC, minimum inhibitory concentration; MIC_50_, MIC value at which growth was inhibited in 50% of isolates; MIC_90_, MIC value at which growth was inhibited in 90% of isolates; S, susceptible; I, intermediate; R, resistant; CTX, cefotaxime; CAZ, ceftazidime; AK, amikacin; GN, gentamicin; CIP, ciprofloxacin; SXT, trimethoprim-sulfamethoxazole.

**Table 3:** Statistical associations between main variables detected in *K. pneumoniae* isolates. Statistical associations were analyzed for the main variables in each group.

Variable	K24 (11)	No-K24 (29)	*p*-Value	OR (CI95%)
*kfuBC*	4	7	- ^§^	
*fyuA*	8	6	0.01	10.22 (2.06–50.76)
Strong/Moderate *	3	11	-	
Weak/NO *	8	18	-	
ST14	3	4	-	
ST45	7	0	0.00	2.75 (1.26–6.01)
*bla*_CTX-M-15_	7	9	-	
*bla*_CTX-M-2_	1	6	-	
*bla*_SHV-5_	1	8	-	
Invasive sample ^‡^	3	11	-	
MDR	9	16	-	
	***kfuBC* (11)**	**No-*kfuBC* (29)**	***p*-value**	**OR (CI95%)**
Strong/Moderate	7	7	0.03	5.50 (1.23–24.50)
Weak/NO	4	22	0.03	0.18 (0.04–0.81)
ST14	5	2	0.01	11.25 (1.75–72.50)
ST45	1	6	-	
*bla*_CTX-M-15_	4	12	-	
*bla*_CTX-M-2_	0	7	-	
*bla*_SHV-5_	3	6	-	
Invasive sample	6	8	-	
MDR	7	18	-	
	***fyuA* (14)**	**No-*fyuA* (26)**	***p*-value**	**OR (CI95%)**
Strong/Moderate	2	12	-	
Weak/NO	12	14	-	
ST14	3	4	-	
ST45	5	2	0.04	6.67 (1.09–40.73)
*bla*_CTX-M-15_	11	5	0.00	15.40 (3.09–76.78)
*bla*_CTX-M-2_	0	7	-	
*bla*_SHV-5_	1	8	-	
Invasive sample	5	9	-	
MDR	11	14	-	
	**Biofilm producer S/M ^#^ (14)**	**No-Biofilm producer S/M ^#^ (26)**	***p*-value**	**OR (CI95%)**
ST14	6	1	0.00	18.75 (1.95–180.00)
ST45	0	7	-	
*bla*_CTX-M-15_	3	13	-	
*bla*_CTX-M-2_	3	4	-	
*bla*_SHV-5_	2	7	-	
Invasive sample	5	9	-	
MDR	8	17	-	
	**ST14 (7)**	**No-ST14 (33)**	***p*-value**	**OR (CI95%)**
*bla*_CTX-M-15_	4	12	-	
*bla*_CTX-M-2_	0	7	-	
*bla*_SHV-5_	0	9	-	
Invasive sample	4	10	-	
MDR	5	20	-	
	**ST45 (7)**	**No-ST45 (33)**	***p*-value**	**OR (CI95%)**
*bla*_CTX-M-15_	5	11	-	
*bla*_CTX-M-2_	1	6	-	
*bla*_SHV-5_	1	8	-	
Invasive sample	2	12	-	
MDR	6	19	-	
	**Invasive sample (14)**	**No-Invasive sample (26)**	***p*-value**	**OR (CI95%)**
*bla*_CTX-M-15_	8	8	-	
*bla*_CTX-M-2_	1	6	-	
*bla*_SHV-5_	2	7	-	
MDR	11	14	-	
	**MDR (25)**	**No-MDR (15)**	***p*-value**	**OR (CI95%)**
*bla*_CTX-M-15_	16	0	0.00	2.78 (1.65–4.68)
*bla*_CTX-M-2_	1	6	0.01	0.06 (0.01–0.59)
*bla*_SHV-5_	4	5	-	

^§^ Non-significance. * Biofilm producer. ^‡^ Invasive sample: blood, cerebrospinal fluid, or synovial fluid. ^#^ S/M: strong/moderate. Abbreviations: MDR, multi-drug resistance.

## Data Availability

All data are available in the manuscript.
